# Banxia Baizhu Tianma Tang decoction and modified Taohong Siwu combined with Western medicine to treat a patient with severe stenosis of the middle cerebral artery: A case report

**DOI:** 10.1097/MD.0000000000036949

**Published:** 2024-01-19

**Authors:** Lingfeng Shu, Qinghai Dai, Pengcheng Zhu, Hongtu Tan, Tao Wu

**Affiliations:** a The First Clinical Medical College of Henan University of Chinese Medicine, Henan, China; b Department of Interventional Medicine, Encephalopathy Center, First Affiliated Hospital of Henan University of Chinese Medicine, Henan, China.

**Keywords:** Banxia Baizhu Tianma decoction, middle cerebral artery, stenosis, stroke, Taohong Siwu decoction

## Abstract

**Rationale:**

Intracranial artery stenosis is an important cause of ischemic stroke, and MCA is one of the most common vessels causing intracranial artery stenosis. At present, there are 3 main treatments for MCA stenosis: medical drug therapy, surgery, and endovascular interventional therapy.

**Patient concerns:**

We report a patient with severe middle cerebral artery stenosis, including his imaging and clinical manifestations.

**Diagnosis:**

Severe stenosis of middle cerebral artery.

**Interventions:**

Banxia Baizhu Tianma decoction combined with Taohong Siwu decoction combined with western medicine.

**Outcomes:**

The stenosis of M1 segment of middle cerebral artery was significantly improved, the stenosis rate was reduced from 70% to 30%, and the clinical symptoms of the patients basically disappeared.

**Lessons:**

Banxia Baizhu Tianma decoction combined with Taohong Siwu plus subtraction combined with western medicine is effective in the treatment of middle cerebral artery stenosis.

## 1. Introduction

Studies have shown that the annual incidence of stroke in patients with MCA stenosis is about 7.0% to 17.7%.^[1]^ Due to the high disability and mortality rate of acute cerebral infarction caused by middle cerebral artery stenosis, active and effective treatment of intracranial artery stenosis is of great significance for the prevention and treatment of stroke. Studies have shown that clinical intervention in patients with acute cerebral infarction can significantly reduce the rate of disability and mortality and improve the prognosis of patients.^[2]^ Chinese traditional medicine believes that the pathogenesis of ischemic stroke is complex, which is obviously related to qi and blood, diet and emotion. Wind, fire, phlegm and blood stasis are the key factors to induce the disease.

## 2. Case presentation

**Figure 1.**
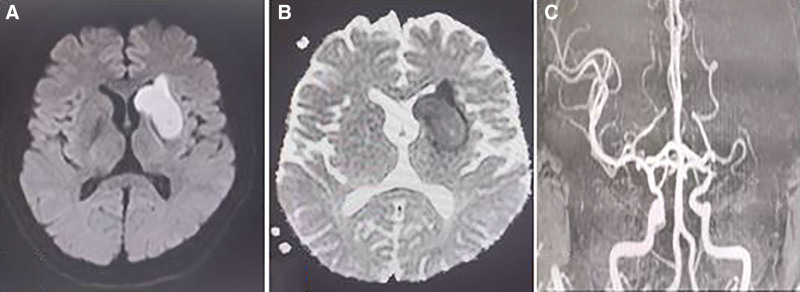
(A and B) Diffusion-weighted imaging of the brain showed fresh infarct focus of left frontal lobe, radiation coronal area, lateral ventricle, basal ganglia, and insular lobe. (C) Head MRA indicates severe stenosis or occlusion of left middle cerebral artery. MRA = magnetic resonance arteriography.

**Figure 2.**
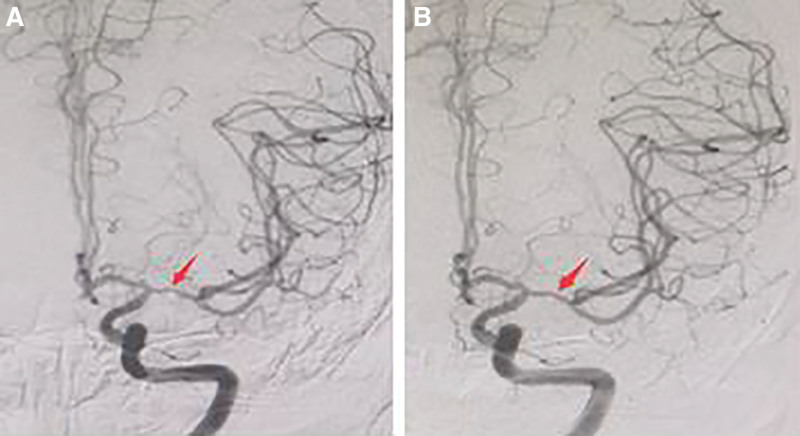
(A) On September 20, 2017, DSA showed that the M1 segment of the left middle cerebral artery was slender, with a stenosis of about 70%. (B) On July 17, 2018, DSA showed a significant reduction in the stenosis of the M1 segment of the left middle cerebral artery, with a stenosis of about 30%. DSA = digital subtraction angiography.

The male patient, 51 years old, was admitted to hospital mainly because of “skewed quarrel, adverse right limb movement for 30 hours, aggravation with unclear speech for 6 hours.” He had a history of diabetes and hypertension. Physical examination: the muscle strength of the right upper and lower extremities was grade IV, and the NIHSS score was 10 points. Head computed tomography examination at the local hospital before admission showed cerebral infarction in the left basal ganglia and calcification in the siphon part of the bilateral internal carotid artery. After admission, diffusion-weighted imaging of the brain showed fresh infarctions in the left frontal lobe, radiation coronal area, paraventricular area, basal ganglia and insular lobe (Fig. 1A and B), and intracranial arteriosclerotic changes and severe stenosis or occlusion of the left middle cerebral artery on magnetic resonance arteriography (Fig. 1C). Combined with the patient’s history and imaging features, the diagnosis was as follows: acute cerebral infarction, type 2 diabetes and hypertension. Digital subtraction angiography examination on September 20, 2017 showed that the M1 segment of the left middle cerebral artery was slender and narrowed by about 70% (Fig. 2A). The diagnosis was that the M1 segment of the left middle cerebral artery was narrow. Western medicine treatment: Atto vastatin (Pfizer Pharmaceutical Co., Ltd., approval number: H20051407), aspirin (Shenyang Kangzhi Pharmaceutical Co., Ltd., approval number: H20103712) combined with clopidogrel (SanofiWinthropIndustrie, approval number: H20171238), and Edaravone (Kunming Jida Pharmaceutical Co., Ltd., approval number: H20080495). Medication: Atto vastatin, oral, 10 mg, once a day. Aspirin, oral, 100 mg once a day. Clopidogrel, orally, 75 mg once a day. Edaravone intravenous drip, 30 mg Edaravone injection was added to 100 mL 0.9% sodium chloride injection, intravenous drip, twice a day. Traditional Chinese medicine treatment: Banxia Baizhu Tianma decoction and Taohong Siwu decoction were added and subtracted. The specific drugs were Qingxia 10 g, Atractylodes macrocephala 20 g, Gastrodia elata 15 g, Poria 15 g, tangerine peel 12 g, fried peach kernel 20 g, safflower 15 g, Radix Rehmanniae 30 g, Radix Paeoniae Alba 25 g, Angelica 30 g, Chuanxiong 12 g, scorpion 10 g, licorice 10 g, Scorpion 15 g, Glycyrrhiza 10g, Acorus calamus 15 g, decocting juice 200 mL, taking medicine twice a day. After being discharged from the hospital, the patients continued to take the above drugs for 10 months, and the patients were reexamined with clear mind, good spirit, no fatigue, significant improvement in the activity of the right limbs and clear speech. digital subtraction angiography on July 17, 2018 showed that the stenosis of the left middle cerebral artery was significantly improved compared with before, and the stenosis was about 30% (Fig. 2B). After the annual telephone follow-up of the patients, the symptoms improved obviously, there was no recurrence of the disease, and there were no adverse reactions.

## 3. Discussion

The treatment of integrated traditional Chinese and western medicine in this patient can reverse the severe stenosis of middle cerebral artery and relieve the symptoms obviously. Among the patients with cerebrovascular stenosis treated in our hospital, patients with cerebrovascular stenosis treated with western medicine only rarely see vascular reversal, and the improvement is not obvious. Banxia Baizhu Tianma decoction and Taohong Siwu decoction combined with free radical scavenger Edaravone, antiplatelet drug aspirin, clopidogrel and lipid-lowering drug Atto vastatin were used in the treatment of ischemic stroke. It can reduce erythrocyte aggregation, improve blood circulation and protect brain nerves with less adverse reactions and higher safety.^[3]^

Banxia Baizhu Tianma decoction is a prescription for eliminating phlegm in traditional Chinese medicine, which is consistent with the principle of TCM treatment for patients with ischemic stroke. Rhizoma Pinelliae has antitussive and expectorant effects, *Atractylodes macrocephala* combined with *Poria cocos* is used to invigorate the spleen and remove dampness, treat the source of phlegm, modern pharmacological studies have confirmed that they have the effects of anti-inflammation and lipid regulation. *Gastrodia elata* has the effect of calming liver wind and regulating negative qi, modern pharmacological studies have confirmed that it can increase blood flow and reduce vascular resistance.^[4,5]^ Licorice and tangerine peel can regulate qi and spleen, dry dampness and resolve phlegm. Taohong Siwu decoction is one of the important prescriptions for regulating menstruation in traditional Chinese medicine, which is composed of Siwu decoction plus safflower and peach kernel, in which peach kernel and safflower have the effect of promoting blood circulation and removing blood stasis. *Angelica sinensis* and Chuanxiong have the effect of descending blood gas. Scorpion and calamus calamus have the effect of regulating qi and promoting blood circulation, dispersing wind and removing dampness, and the whole prescription has the effect of nourishing blood and promoting blood circulation. Modern pharmacology has proved that Taohong Siwu decoction can promote fibrinolysis and improve microcirculation.^[6]^

## 4. Conclusion

Banxia Baizhu Tianma decoction combined with Taohong Siwu plus subtraction combined with western medicine is effective in the treatment of middle cerebral artery stenosis. Compared with surgery, endovascular intervention and other treatment methods, it not only saves the operation cost for patients, but also avoids a series of postoperative complications, effectively improves the ability of daily life of patients, and improves the quality of life of patients. It can be used as the first choice for clinical treatment of patients with intracranial artery stenosis at the present stage, and it has significant clinical application value.

## Author contributions

**Conceptualization:** Tao Wu.

**Data curation:** Pengcheng Zhu.

**Formal analysis:** Hongtu Tan.

**Investigation:** Qinghai Dai.

**Writing – original draft:** Lingfeng Shu.

**Writing – review & editing:** Tao Wu.
